# Multipath Identification and Mitigation for Enhanced GNSS Positioning in Urban Environments

**DOI:** 10.3390/s25196061

**Published:** 2025-10-02

**Authors:** Qianxia Li, Xue Hou, Yuanbin Ye, Wenfeng Zhang, Qingsong Li, Yuezhen Cai

**Affiliations:** 1 Surveying and Mapping Institute Lands and Resource Department Guangdong Province, Guangzhou 510500, China; liqianx5@mail.sysu.edu.cn (Q.L.); yeyuanbin2025@163.com (Y.Y.); zhangwenfeng_2025@163.com (W.Z.); 2 School of Geography and Planning, Sun Yat-sen University, Guangzhou 510006, China; caiyzh5@mail2.sysu.edu.cn; 3 Guangdong Hydropower Planning & Design Institute Co., Ltd., Guangzhou 510635, China; li.qs@gpdiwe.com

**Keywords:** GNSS, urban environment, multipath, wavelet transform, frequency spectrum

## Abstract

Due to the increasing demand for accurate and robust GNSS positioning for location-based services (LBS) in urban regions, the impacts prevalent in metropolitan areas, like multipath reflections and various interferences, have become persistent challenges. Consequently, developing effective strategies to address these sophisticated influences has become both a primary research focus and a shared priority. In this paper, the authors explore an approach to identify and mitigate the drawbacks arising from multipath effects in urban positioning. Unlike conventional ways for building complex models, an adaptive data-driven methodology is proposed to identify the fingerprints of a multipath in GNSS observations. This approach utilizes the Fourier transform (FT) to examine code multipath and other error sources in terms of frequency, as represented by the power spectrum. Wavelet decomposition and signal spectrum methods are subsequently applied to seek traces of code multipath in multilayer decompositions. Based on the exhibited multipath features, the impacts of multipath in GNSS observations are detected and mitigated in the reconstructed observations. The proposed method is validated for both static and dynamic positioning scenarios, demonstrating seamless integration with existing positioning models. The feasibility has been verified through a series of experiments and tests under urban environments using navigation terminals and smartphones.

## 1. Introduction

Since the advent of the Global Positioning System (GPS) in the 1990s, satellite positioning has been rapidly adopted across various fields and by individual users. To meet the growing need for accurate performance, multiple augmentation infrastructures have been established, including differential base stations, continuously operating reference systems (CORS) networks, wide-area differential GNSS (WADGNSS), ground-based augmentation systems (GBASs), and satellite-based augmentation systems (SBAS). Collectively, these systems provide both professional and consumer services through differential corrections, delivering specified levels of accuracy and availability [1,2,3]. In mass-market domains, such as logistics and supply chains, precision agriculture, healthcare, and related location-based services, navigation terminals such as tablets and personal digital assistants (PDAs) have become increasingly prevalent. With rapid advances in mobile hardware and software, smartphones and tablets have become mainstream positioning devices due to their ubiquity, portability, and affordability. Since Google enabled access to raw GNSS measurements on Android devices in 2016, end users have been able to gain access to pseudorange, carrier phase, and navigation messages [4,5]. This provides smartphone and tablet users with the opportunity to develop more flexible algorithms that combine with external corrections and multi-source information to enhance the accuracy and reliability of GNSS positioning, which was previously only available for geodetic receivers [6,7,8].

Growing demands for stringent positioning performance and intelligent positioning, navigation, and timing (PNT) services have driven advancements in user terminal technology. These demands have also spurred the development of extensive augmented positioning infrastructure [9]. To achieve improved positioning performance using portable and low-cost terminals, it is still necessary to address some persistent challenges that hinder tablets and smartphones from achieving satisfactory accuracy and reliability [10]. This notorious influence would endanger positioning under urban conditions and metropolitan avenues, since most human activities and high-value economic operations take place within these areas [11]. Among various error sources affecting urban localization, multipath remains unresolved and is mostly incurable, as it cannot be perfectly modeled or mitigated using conventional approaches [12].

Signals reaching the receiver antenna directly from satellites are referred to as line-of-sight (LOS) signals. A multipath occurs when LOS signals are blocked by terrain or obstacles. Reflection, scattering, and diffraction can interfere with LOS signals before antenna reception. The composite received signal thus contains both LOS components and multipath contaminants, forming non-line-of-sight (NLOS) signals [13]. A multipath can cause the correlation peak in the receiver correlator to become skewed, resulting in delays to LOS, signal strength attenuation, and finally upsetting the range to the satellite measured by the receiver correlator [14].

For a geodetic receiver, there are several ways to improve the code tracking loop and carrier tracking loop. Narrow correlation technique, code correlation reference waveforms technology, and double delta technology are typical examples of hardware techniques [15]. For antenna designs, developments have covered polarized antennas [16], the ground plate approach [17], choke ring designs [18], antenna arrays [19], and digital beaming steering techniques. These techniques have obviously curbed the impacts of multipath to a lower level. Alternatively, multipath resilience can be addressed through the innovative design of navigation signals. So far, two main signal modulation patterns, binary phase shift keying (BPSK) and binary offset carrier (BOC), have been adopted. The latter was proposed by John W. Betz in 2001 to achieve signal spectrum separation and mitigate multipath, as the auto-correlation function can form a narrower main peak, which in turn results in better ranging performance and stronger anti-interference ability than conventional BPSK modulation [20]. The BOC has become a new signal system and is widely used in GNSS.

Despite significant advancements in GNSS hardware and antenna design, multipath effects remain fundamentally challenging, and it is difficult to mitigate this influence in complex urban environments due to the persistent complexities of signal propagation [21]. The final way to cope with multipath is advanced data processing and algorithmic enhancements. As a sophisticated interference source, multipath exhibits dynamic variations in power, propagation delay, amplitude, phase, and frequency. Beyond hardware and antenna improvements, researchers employ diverse physical modeling and mathematical techniques to counteract these effects [21]. According to the physical ray tracing method, the multipath signal is simulated by combining a propagation model and an attenuation model, which proves inadequate for real-world scenarios due to the inherent complexity of reflected, diffracted, and scattered signals [22]. Based on the repeatability of satellite orbits and the integrity of surrounding environments, multipath can be mitigated using an empirical filtering method [23]. This scheme is not suitable for covering dynamical applications, as they require prior knowledge and data. A similar dilemma exists in the spherical and harmonic model, where multipath corrections are computed based on satellite elevation and azimuth [24]. To overcome these limitations, numerous efforts have been made in processing observation data, particularly in terms of spectrum and filtering. The conventional Fourier transform (FT) can exhibit features of a multipath signal, with some frequencies contained in the observation data series. As multipath is usually regarded as a nonstationary signal, the short-time Fourier transform (STFT) can discover the time when the multipath occurs locally as an event with a specific frequency. To explore multipath from the mechanism of signal and spectrum, empirical mode decomposition (EMD) is capable of detecting multipath in terms of instant frequency and Hilbert spectrum [25]. Starting from GNSS observations as input, these approaches have evolved into a data-driven system in which multipath can be directly observed in terms of energy, frequency, and spectrum, without the need for simulation and modelling. In contrast to the EMD, which is vulnerable to the mode mixing problem and constrained mathematical framework, the wavelet transform (WT) is a flexible tool that can be dynamically extended to identify instantaneous frequency and temporal features for complex signals, such as multipath or interference [26].

Data processing and algorithm-enhancing methods are the most optimal selection for smartphone receivers to tackle the multipath dilemma, as there is very limited room for smartphones to implement complex hardware and antenna schemes. To achieve a low-cost budget, power saving, and size minimization, smartphones are initially equipped with low-cost, low-power GNSS chipsets, which lead to weak sensitivity during signal lock and a low and irregular signal-to-noise ratio (SNR) [7]. The antenna embedded in the smartphone is a passive, linearly polarized antenna. This antenna is used to jointly receive both GNSS and wireless communication signals. When the circularly polarized GNSS signal is received by a linearly polarized antenna, observations are more vulnerable to multipath and other interferences, especially in highly dynamic and complex urban environments [27]. One more disadvantage is the battery life-saving mechanism. This forces the smartphone components, which have a duty cycle, to periodically switch off and on. This subsequently results in the discontinuity of the carrier phase observation [28]. This, in turn, degrades carrier phase quality and endangers ambiguity convergence for high precision positioning.

Given this context, it is challenging for smartphones to achieve satisfactory positioning performance due to a trade-off in hardware designs. In recent years, scholars and professionals have been seeking data processing techniques and algorithms as remedies for smartphones’ poor suppression capability of multipath and interference [29,30,31,32,33]. To date, these techniques and algorithms can be classified as statistical processing, which is based on temporal correlation, empirical models utilizing features of fixed scenarios and the repeatability of satellite orbits, and filtering approaches in which observations are inspected in terms of data series and frequency spectrum [21].

This paper has made efforts to develop a WT-based scheme into an active, data-driven mode, allowing for the inspection of multipath in a flexible and effective manner, thereby removing it from GNSS observations collected by smartphones. To facilitate convenient and robust positioning in harsh environments, such as complex urban regions and blocked areas, we begin by refining observation views for smartphone data using an adaptive data-driven mode based on temporal and spatial inspection and decomposition processes for observation data sequences. To inspect code multipath and other error sources, the conventional Fourier transform is first employed to display their frequency spectra, and wavelet decomposition is subsequently applied to identify traces of code multipath involved in the observations. Based on the exhibited multipath features, the fingerprints of code multipath in GNSS observations are inspected, and their impact is cancelled in the reconstructed process using a WT. This adaptive scheme is verified to be effective for both static and dynamical positioning modes using smartphone code observations collected under harsh and restricted scenarios. Following the first part of the introduction, code observation expressions and multipath error are outlined in the second part to provide computation using code minus phase combination in the case of single-frequency observations. The third part highlights the feature exploration and reduction of code multipath for smartphone positioning using comprehensive frequency and spectrum approaches, including the WT. The paper presents tests and validations using smartphone observations, where both GPS and BDS signals are collected in urban environments.

## 2. Methodology

Following Section 1, this section, in view of code multipath identification and mitigation based on observation data processing, first reviews the basic method to calculate code multipath with a combination of carrier phase observations. Since some navigation terminals and smartphones cannot provide carrier phase observation data, we developed a method that combines wavelet decomposition with spectrum analysis to flexibly process GNSS observations and identify code multipath signals.

### 2.1. Code Multipath Calculating with Carrier Phase

Starting from basic observations for GNSS signals, if hardware delays, code bias, and frequency bias are ignored, code range and carrier phase observations collected on station *r* for satellite *u* can be described as follows [14]:

(1) $$P_{i,r}^{u}=\rho_{i,r}^{u}+c(\delta t_{r}-\delta t^{u})+I_{i,r}^{u}+T_{r}^{u}+M_{i,r}^{u}+\varepsilon_{P_{i},r}^{u}$$

(2) $$\lambda_{i}\varphi_{i,r}^{u}=\rho_{i,r}^{u}+c(\delta t_{r}-\delta t^{u})-\lambda_{i}N_{r}^{u}-I_{i,r}^{u}+T_{r}^{u}+m_{i,r}^{u}+\varepsilon_{\varphi_{i,r}}^{u}$$

where ρ is the geometric range, φ is the carrier phase; i is the index of frequency; c is the speed of light; $\delta t_{r}$ and $\delta t^{u}$ are the receiver clock error and satellite clock error, respectively; I is the ionospheric delay, T is the tropospheric delay; $\lambda_{i}$ is the wavelength of frequency $f_{i}$, and N is the initial ambiguity; M and m are the multipath errors in code range and carrier phase observations, respectively; ε is the observation noise.

In cases where dual-frequency phase observations are available and their propagation paths are the same through the atmosphere, with the removal of $I_{i}$ based on the fusion of carrier phase observations, the following combination can be derived [34]:

(3) $$M_{i}=P_{i}-\frac{f_{i}^{2}+f_{j}^{2}}{f_{i}^{2}-f_{j}^{2}}\lambda_{i}\varphi_{i}+\frac{2f_{j}^{2}}{f_{i}^{2}-f_{j}^{2}}\lambda_{j}\varphi_{j}+k$$

This equation refers to the code multipath as the combination of code range and phase observations for a dual-frequency GNSS receiver. $f_{i}$ and $f_{j}$ represent the frequencies for two carrier phases, respectively. k is the function of $N_{i}$, $N_{j}$, $m_{i}$, $m_{j}$, and carrier phase observation noise ε of the receiver. Multipath for phase observation is considered to be very small compared to that for code ranges and can therefore be ignored [35]. Meanwhile, observation noise is not taken into consideration. When no cycle slips exist or they have been successfully recovered, the function k becomes constant and can be derived by averaging over the observation time span.

From code and phase expressions (1) and (2), we have the following combination for code minus phase (CMP):

(4) $$\begin{array}{lll} P_{i}-\lambda_{i}\varphi_{i} & =2I_{i}+M_{i}+\lambda_{i}N_{i}-m_{i}+\varepsilon_{P_{i}\varphi }\\ & =2I_{i}+M_{i}+\lambda_{i}N_{i}+k^{\prime } & \end{array}$$

where $k^{\prime }$ indicates carrier multipath, code, and carrier noise. In this expression, code multipath is combined with twice the ionosphere delay, carrier phase ambiguity, code noise, and carrier noise, since carrier noise is very small and can be ignored in contrast to code multipath.

The current approach to derive code multipath approximation by Formula (4) assumes that ionosphere delay is steady within a short time span, say 10 min or 15 min, or observation is conducted in a region where ionosphere activity is relatively steady and can be treated as a linear variation that can be eliminated or fitted out by average or linear detrend processing. For observation spans longer than 15 min, we can compensate for ionosphere delay using broadcast parameters. An alternative approach is to separate the observation data spans when the observation time is long or the ionosphere is active, thereby ensuring that derived results are not degraded [36,37].

### 2.2. Differential Code Ranges Between Satellites

As previously stated, several drawbacks are associated with the smartphone carrier phase, leading to gradual error accumulation, discontinuity, low quality, and frequent cycle slips, which make it challenging to investigate code multipath with the aid of carrier phase. To facilitate code multipath inspection without the aid of carrier phase observation, differential code ranges between satellites (DRS) are formed to reduce the influences of both receiver clock bias and most atmospheric delays. Following Equation (1), DRS between satellite u and v can be expressed as

(5) $$\Delta P_{i,r}^{uv}=\Delta \rho_{i,r}^{uv}+c\cdot \Delta \delta t^{uv}+\Delta I_{i,r}^{uv}+\Delta T_{r}^{uv}+\Delta M_{i,r}^{uv}+\Delta \varepsilon_{P_{i},r}^{uv},$$

Since receiver clock bias and hardware delays are removed in DRS, if code bias and receiver channel bias are ignored, we have differential atmospheric delay $\Delta I_{i,r}^{uv}$ and $\Delta T_{r}^{uv}$, differential satellite clock bias $\Delta \delta t^{uv}$, differential code multipath $\Delta M_{i,r}^{uv}$, and differential observation noise $\Delta \varepsilon_{P_{i},r}^{uv}$ in DRS data series. As a general rule, the satellite with the highest elevation is usually selected as the reference code range, and the differential multipath $\Delta M_{i,r}^{uv}$ mainly reveals the multipath value of satellite v relative to the reference satellite *u*.

### 2.3. Wavelet Transform for Multipath Detection

Following the FT and STFT, the WT was first introduced by J. Morlet in 1974 due to the need for analyzing nonstationary and nonlinear signals and data sequences [38]. The WT was then systematically established and developed by Y. Meyer, S. Mallat, and many other scholars and professionals [39,40]. To extensively inspect a signal or function s(t) from aspects of both time and spectrum, the WT maps s(t) into time and scale spaces that form a two-dimensional domain [41]. This mapping is denoted by $W_{s}(a,b)$:

(6) $$W_{s}(a,b)=\frac{1}{\sqrt{a}}\int_{-\infty }^{+\infty }s(t)h^{*}(\frac{t-b}{a})dt=\int_{-\infty }^{+\infty }s(t)h_{ab}^{*}(t)dt,$$

where $h^{*}(t)$ is the conjugate function of h(t), and h(t) is known as the mother wavelet, which can generate and form basic functions of the transform. These derived functions are usually called daughter wavelets and given by

(7) $$h_{ab}(t)=\frac{1}{\sqrt{a}}h(\frac{t-b}{a}),$$

Formula (6) expresses the flexible expansion of the signal s(t) controlled by the selected function h(t) and adjustable variables a and b in daughter wavelets. In contrast to the STFT, the parameters a and b in deriving wavelets provide multi-scale and dynamic inspection windows, enabling the flexible exploration of the complex signal being analyzed. During the signal decomposition into multiple layers or windows, it can be compressed or dilated by setting the scaling parameter a to a proper value. The adjustment of the shift parameter b can form a variable temporal translation for the observing point of the complex signal. This mechanism will bring great convenience for the exploration and discovery of code multipath as it is hidden in GNSS observations, together with other sources of errors and blunders.

The extraction of the desired component from a complex signal can be achieved through reconstruction using wavelet coefficients $W_{s}(a,b)$. The inverse WT is expressed as

(8) $$s(t)=\frac{1}{c}\int_{-\infty }^{+\infty }\int_{0}^{+\infty }W_{s}(a,b)h(\frac{t-b}{a})\frac{da}{a^{2}}dt,$$

When the signal is decomposed based on predefined frequency features, it is split into different layers of higher-frequency and lower-frequency components. The desired signal, such as code multipath and other error events, is typically exhibited as high-pass signals among the decomposed layers. It can be extracted or reconstructed by analyzing its frequency signature and identifying layer sources through wavelet functions and their decomposed coefficients.

**Figure 1:**
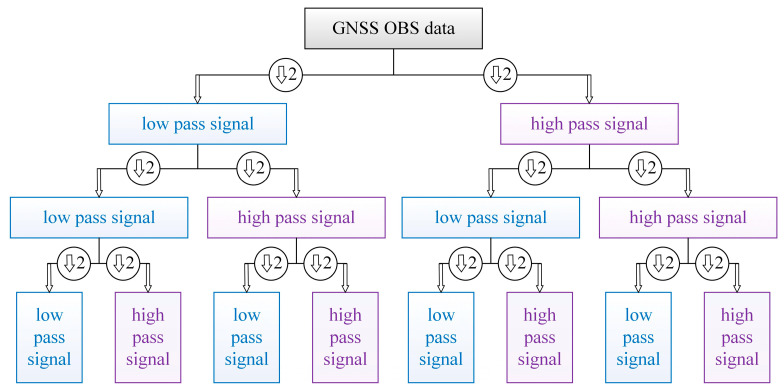
Multiresolution wavelet packet algorithm with 3 levels.

In practical applications, the discrete wavelet transform (DWT) is performed through complete wavelet packet decomposition, which is well-localized and adaptively partitioned in both time and frequency scales. The dyadic multiresolution decomposition can be illustrated by Figure 1, which uses three levels of filtering layers as an example.

**Figure 2:**
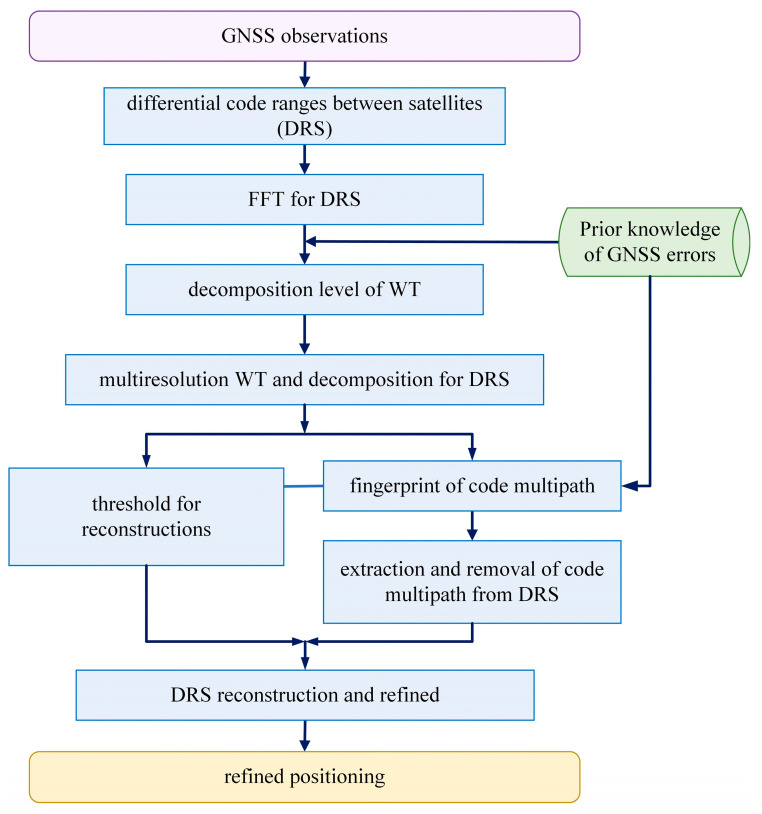
The basic flows of multipath inspections, extractions and eliminations using WT.

To cancel the influence of receiver clock bias, the mode of differential process between satellites is adopted in positioning. As indicated by Equation (5), the differential range of satellites (DRS) contains relative code multipath, which affects the result of positioning. Through the combined WT and FT, multipath can be identified and extracted from the DRS observation series, as shown in Figure 2.

After the FFT is performed in DRS, we can obtain a rough relative frequency distribution for each component, including differential code ranges, ionosphere and troposphere delays, code multipath, relative satellite clock biases, and observation noise. When the satellite clock is corrected by the ephemeris, the remaining error is approximately one nanosecond and typically appears as a component with a relatively higher frequency than code multipath. The observation noise has the highest frequency in the distributions. The frequency of code multipath is expected to be higher than the gradually changing ionosphere delay and lower than the observation noise, as well as part of possible multipath diffractions. In Equation (5), the differential code range is the slowest-changing component, representing the gradual relative motions of the differential satellites. These distributions will help us to plan WT decomposition levels. In practice, more levels (say 10 levels) will be selected to exhibit more specific and detailed components, such as possible diffractions, overlapping of code multipath and ionosphere delays, or potential radio interferences.

When DRS is decomposed into different layers according to the selected levels, we can extract code multipath and reconstruct optimal DRS observations by removing or reducing undesired components. Since each layer is defined by WT coefficients, we can rebuild the expected component (e.g., code multipath) using WT coefficients for the related layer or layers. When the component is distributed across several levels, the threshold controls the extent to which each layer is selected. A threshold value of 1 indicates full selection of this layer, while 0 indicates complete rejection. A weighted threshold value means a partial selection and is usually determined by residuals from adjustment or fitting. This was not used in our test, as it makes the process more complex.

## 3. Results and Analysis

**Figure 3:**
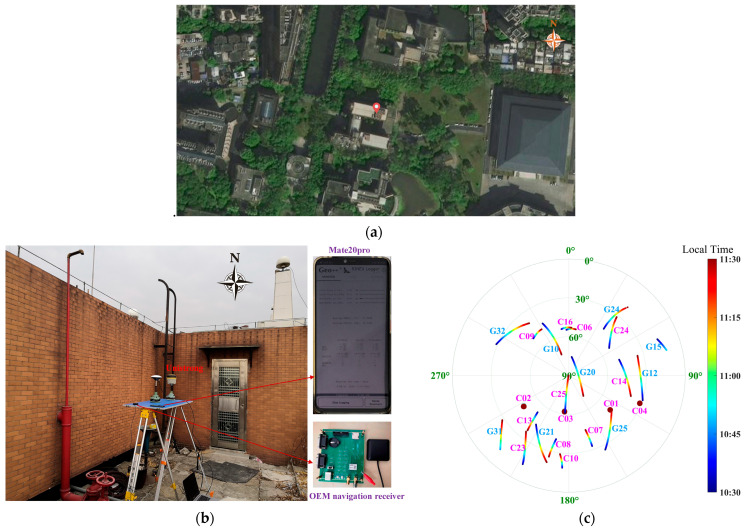
Data collection for static test. (**a**) The location of antennas at the red point; (**b**) the scenario; (**c**) sky plot of satellite trajectories.

In order to reach an understanding of code multipath and the subsequent analysis of its impact on positioning, a dual-frequency geodetic receiver, a single-frequency OEM navigation receiver, and a smartphone, the Huawei Mate 20 Pro, were mounted on the top floor of the geographic building of the basic campus at Sun Yat-sen University for data collection on 14 January 2020. The red point in Figure 3a is the location of the antennas. As shown in Figure 3b, there are concrete walls near the receivers to the west and north. These obstacles are likely to cut off satellite signals and cause reflections or diffractions. They are the main sources of multipath in GNSS positioning. A sky plot of satellite trajectories is shown in Figure 3c. In the north and west, there are few satellites due to the barrier of the walls.

### 3.1. Investigation of Code Multipath

**Figure 4:**
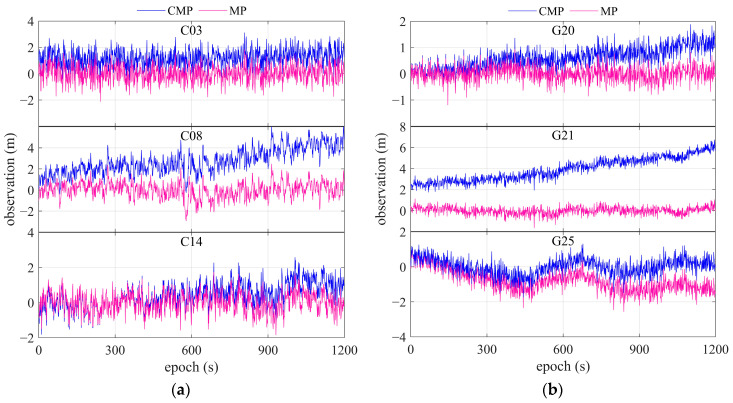
CMP and multipath derived from dual-frequency receiver for (**a**) BDS satellites C03, C08, and C14 and (**b**) GPS satellites G20, G21, and G25.

**Figure 5:**
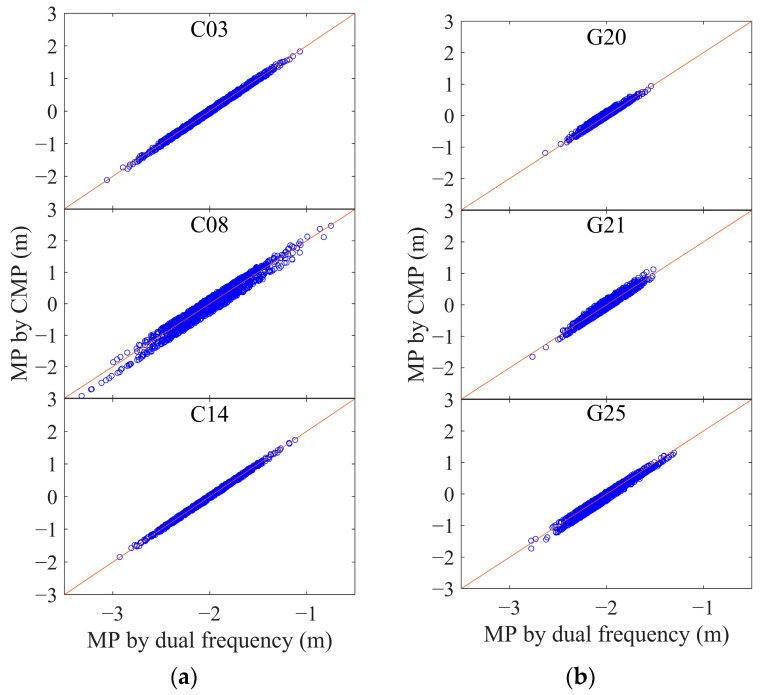
Relationships and differences between results of code multipath derived by the CMP and the combination of dual-frequency observations. The red line is the function of y = x, and the blue points show the code multipath obtained using the two approaches in the x and y directions, respectively. (**a**) BDS satellites C03, C08, and C14; (**b**) GPS satellites G20, G21, and G25.

With the assistance of carrier phase, the multipath can be obtained using a dual-frequency combination and CMP, as shown in Equations (3) and (4), respectively. Figure 4 shows the code multipath in magenta, as observed using Equation (3), and the CMP in blue for the dual-frequency geodetic receiver. Figure 5 illustrates the relationship between the multipath derived from the CMP and the dual-frequency combination equation. From the indicated differences, the results obtained using the two approaches for code multipath are basically equivalent.

**Figure 6:**
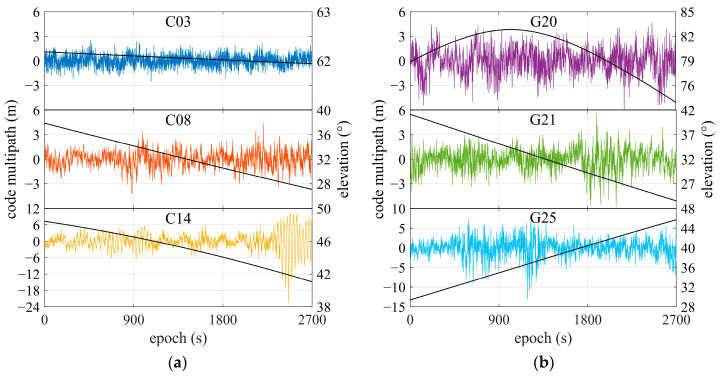
Results of code multipath and elevations of some satellites. The colorful lines represent the multipath of satellites, and black lines represent the elevation. (**a**) BDS satellites C03, C08, and C14; (**b**) GPS satellites G20, G21, and G25.

Figure 6 shows the code multipath result and satellite elevation for the single-frequency OEM navigation receiver. During the collection of observation data, elevations for all satellites were larger than 20°. In Figure 6a, C03 and C08 are the GEO and IGSO satellites. There are no obvious changes in the code multipath values, as these satellites are moving slowly with relatively high elevations. The multipaths of the G25 satellite and C14 are somewhat larger, even though they have higher elevation angles, since their signals have suffered multipath reflections from surrounding walls, which are indicated by the azimuths and elevations shown in Figure 3. It is noted that the G20 is moving from north to southeast with a higher elevation, and its signal is meeting reflections from two walls that are vertical to each other and facing east and north, respectively.

**Figure 7:**
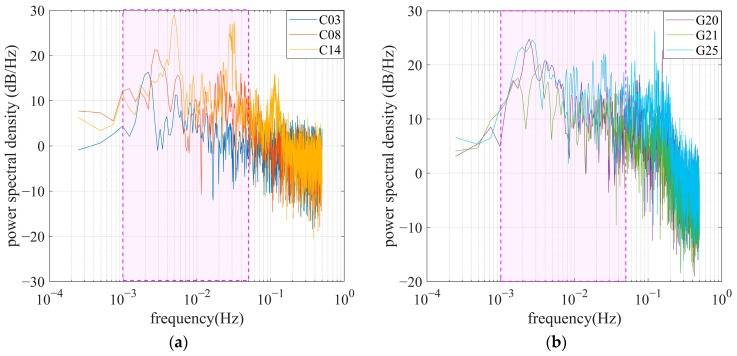
Power spectral density of code multipath for observations collected by single-frequency OEM navigation receiver. The pink area within the dashed box indicates the primary frequency components of the code multipath. (**a**) BDS satellites C03, C08, and C14; (**b**) GPS satellites G20, G21, and G25.

To enable multipath identification and mitigation using a WT-based approach, periodic spectrum estimation was performed to obtain the power spectrum and frequency features of the multipath. Figure 7 represents the power spectral density (PSD) of the multipath shown in Figure 6. It can be found that the central frequency of code multipath is mainly distributed in the range of 1~50 MHz, as indicated by the magenta rectangle. The PSD values rising above about 0.1 Hz can be attributed to the noise effect in the observations collected by the OEM navigation receiver.

**Figure 8:**
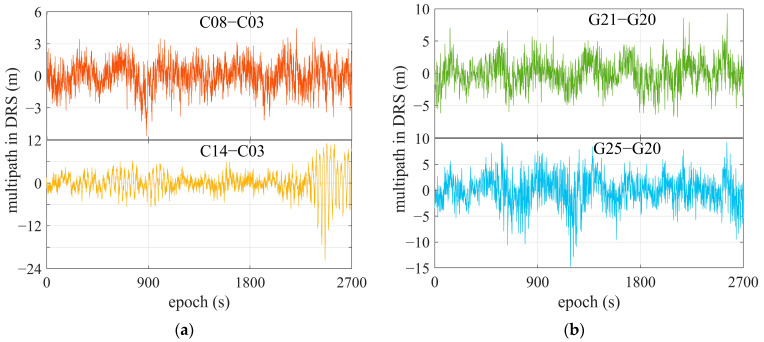
Satellite differential code multipath of some satellites. (**a**) C08 (red line) and C14 (yellow line), referring to C03; (**b**) G21 (green line) and G25 (blue line), referring to G20.

**Figure 9:**
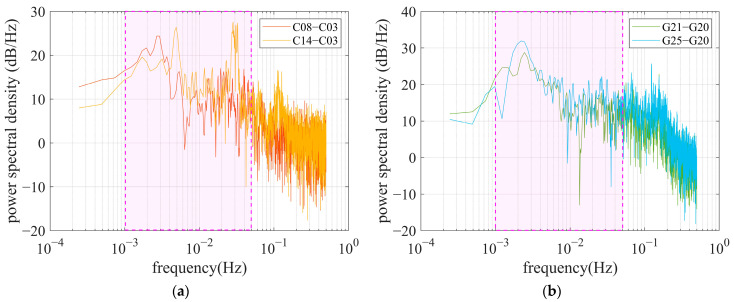
Power spectral density of satellite differential code multipath. The pink area within the dashed box indicates the primary frequency components of the satellite differential code multipath. (**a**) C08 (red line) and C14 (yellow line), referring to C03; (**b**) G21 (green line) and G25 (blue line), referring to G20.

To seek a mechanism for multipath inspection using a comprehensive spectrum, we also investigated the frequency of code multipath $\Delta M_{i,r}^{uv}$ in DRS. Based on the code multipath in Figure 6, the satellite differential code multipath in DRS can be calculated and is shown in Figure 8, where C03 and G20, with high elevations, are selected as reference satellites. Figure 9 gives the corresponding power spectral densities for code multipath in the DRS series. As the multipath for satellites with high elevation is relatively weak, the multipath results in DRS mainly exhibiting multipath impacts suffered by satellite signals with lower elevations. It can be found that the power spectral densities of code multipaths in DRS are similar to those revealed in Figure 7.

### 3.2. Multipath Identification with Wavelet Transform

Based on the technical framework introduced previously, this section explores GNSS code multipath using the WT decomposition of combined GNSS observations and reconstructs refined observations to improve positioning accuracy. To pave the way for code multipath inspection and mitigation in smartphone receivers, DRS is used as input for WT, eliminating the need for carrier phase. Multipath is inspected among the WT decomposed layers, in combination with assist frequency and power spectrum information. Finally, code multipath is extracted using related decomposition layers and removed from the DRS observation for improved positioning.

#### 3.2.1. Multipath Inspection for OEM Navigation Receiver

**Figure 10:**
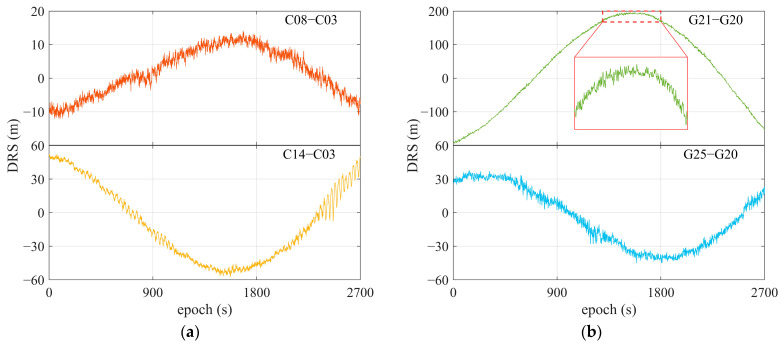
DRS of some satellites using single-frequency OEM navigation receiver. (**a**) C08 and C14, referring to C03; (**b**) G21 and G25, referring to G20. The area within the solid red rectangle shows a zoomed-in view of the region inside the dashed red rectangle.

Figure 10 gives DRS for satellites C08 and C14, with C03 as reference, and G21 and G25, with G20 as reference. Data were collected under the scenarios depicted in Figure 3, using a single-frequency OEM navigation receiver.

**Figure 11:**
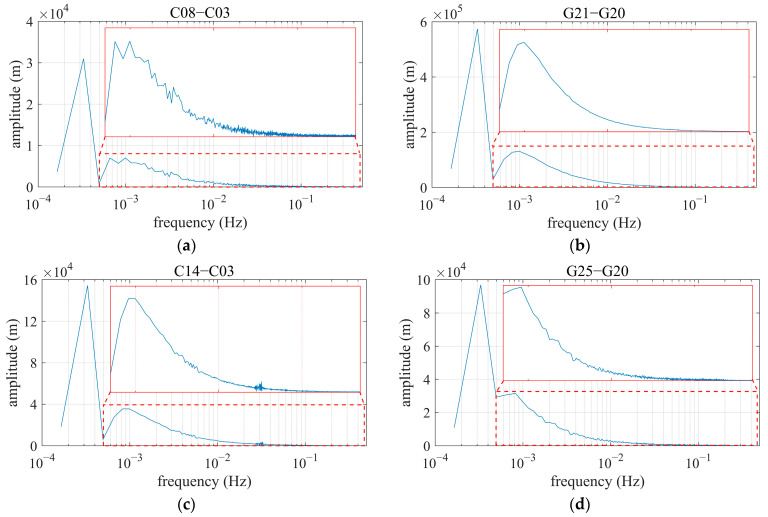
FFT frequency spectrum of DRS, based on single-frequency OEM navigation receiver. In each subfigure, the blue line is the spectrum of DRS, and the area within the solid red rectangle shows a zoomed-in view of the region inside the dashed red rectangle. (**a**) C08, referring to C03; (**b**) G21, referring to G20; (**c**) C14, referring to C03; (**d**) G25, referring to G20.

Figure 11 depicts the corresponding frequency spans contained in DRS observations shown in Figure 10. Under the designed test scenarios in Figure 3, the main amplitude peak corresponds to the central frequencies of DRS observations, with values ranging from 2 × 10^−4^ Hz to 5 × 10^−4^ Hz, which is the lower part of the frequency span and exhibits spatial differential changes in relative code ranges. The frequencies of relative ionospheric and tropospheric delays are also involved in these spans. The subpeaks of amplitude, highlighted by red windows in the subplots, primarily represent the frequencies of DRS code multipaths, with values around 10^−3^ Hz. The rest of the part contained in DRS is the relative satellite clock biases. After correction using the broadcast ephemeris, the remaining residuals are approximately 2 nanoseconds. This means that their amplitudes are smaller, but their frequencies are higher than those of DRS code multipaths and are believed to couple with the noise of DRS code ranges.

**Figure 12:**
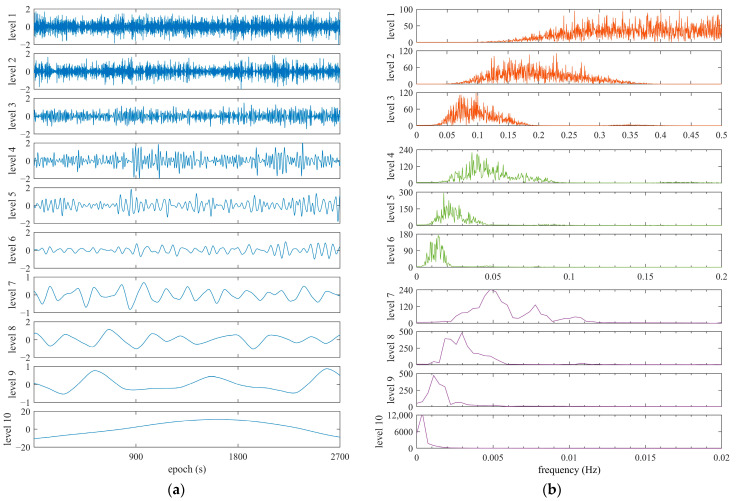
Decomposition layers and frequency spectrums of DRS using WT for BDS C08, referring to C03, collected by OEM navigation receiver. (**a**) Decomposition level; (**b**) frequency spectrum.

**Figure 13:**
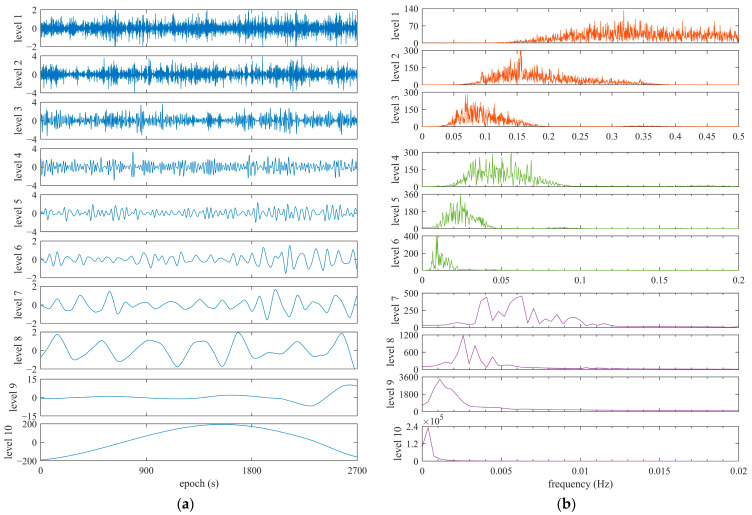
Decomposition layers and frequency spectrums of DRS using WT for GPS G21, referring to G20, collected by OEM navigation receiver. (**a**) Decomposition level; (**b**) frequency spectrum.

**Figure 14:**
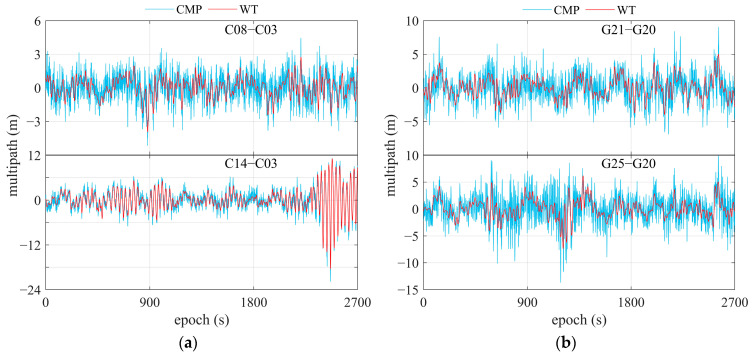
Comparison of the multipath extracted from DRS by WT and that obtained by CMP. (**a**) C08 and C14, referring to C03; (**b**) G21 and G25, referring to G20.

Figure 12 and Figure 13 show the given WT-based decomposition layers and their frequency spectrums using the BDS C08 satellite and GPS G21 as examples, which were collected by the OEM navigation receiver. DRS was decomposed into 10 layers based on prior information on frequency and power spectrum features. From the results presented in Figure 12 and Figure 13, fingerprints of code multipath can be searched and explored according to the frequency and power spectrum cues obtained in the previous subsection. Taking the G21 satellite, for instance, it is believed that layers 4 to 8 are related to code multipath, and it is extracted using these layers. The results of code multipath are presented in Figure 14, in comparison with those computed using Equation (4). We can see that observation noise is contained in the code multipath obtained using Equation (4), but the noise is removed when WT is employed in this method. This comparison verifies that the extraction of code multipath from DRS is consistent with the approach given by Equation (4).

#### 3.2.2. Multipath Inspection for Smartphone

**Figure 15:**
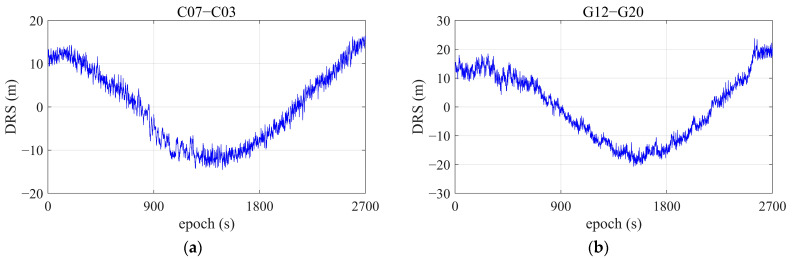
DRS of some satellites, using the Huawei Mate 20 Pro smartphone. (**a**) C07, referring to C03; (**b**) G12, referring to G20.

Given the studies in the previous section, the purpose of this research is to further explore code multipath detection and elimination for smartphone receivers using a comprehensive approach based on the WT. Tests and data collections were conducted using a Huawei Mate 20 Pro under the scenarios presented in Figure 3. For smartphone positioning in metropolitan areas, GNSS signals are often blocked and disrupted by building walls and dense vegetation, such as forests. Under normal conditions in urban streets, GNSS observations covering a continuous 45-min time span were selected for analysis. Figure 15 shows the DRS observation data of BDS satellite C07 and GPS satellite G12, for example.

**Figure 16:**
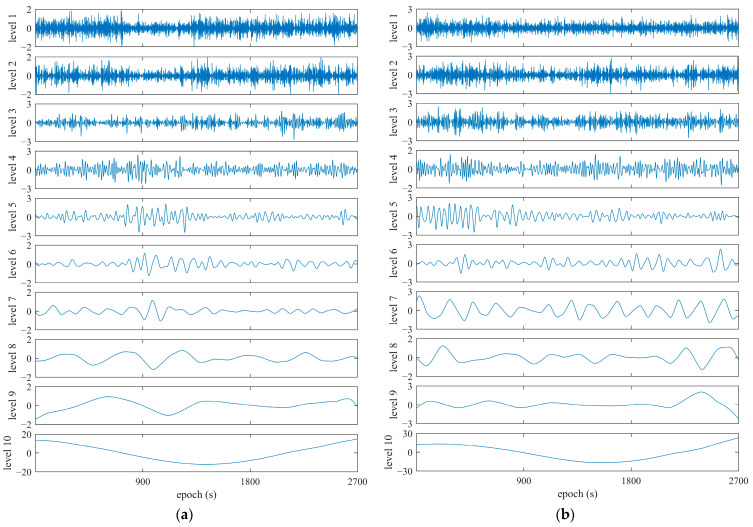
Decomposition of DRS with WT for satellites, using the Huawei Mate 20 Pro smart phone. (**a**) C07, referring to C03; (**b**) G12, referring to G20.

Following the WT-based principle, we decomposed the DRS for satellites observed during the test under urban conditions. To properly dispose of multipath fingerprints in DSR data series, decomposition was performed in the form of 10 layers for DRS observations. Figure 16 shows the decomposition of DRS with the WT for satellites C07 and G12.

**Figure 17:**
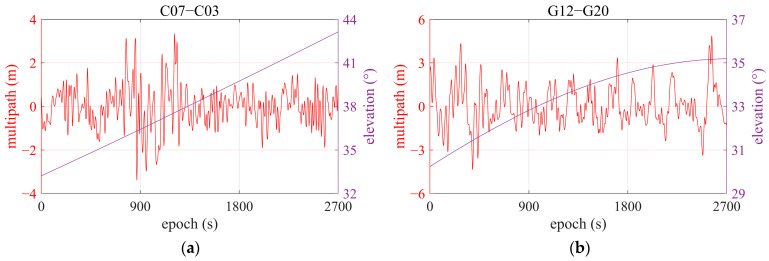
Multipath extracted from DRS by WT. (**a**) C07, referring to C03; (**b**) G12, referring to G20.

Figure 17 shows the satellite differential code multipath results for satellites C07 and G12, which correspond to C03 and G20, respectively, and were extracted using a WT-based comprehensive frame. Since elevations of both satellites are higher than 30°, the multipath is not larger than 3 m. C07 exhibits some notable changes around 750–1250 epochs, possibly due to its location southeast of the receiver, where its signal may be reflected by the walls in both the north and west.

### 3.3. Positioning Test and Validation

To further validate the feasibility of the proposed technical framework, GNSS positioning tests and experiments were conducted in urban scenarios. The tests were conducted under both static and kinematic positioning conditions.

#### 3.3.1. Static Positioning

**Figure 18:**
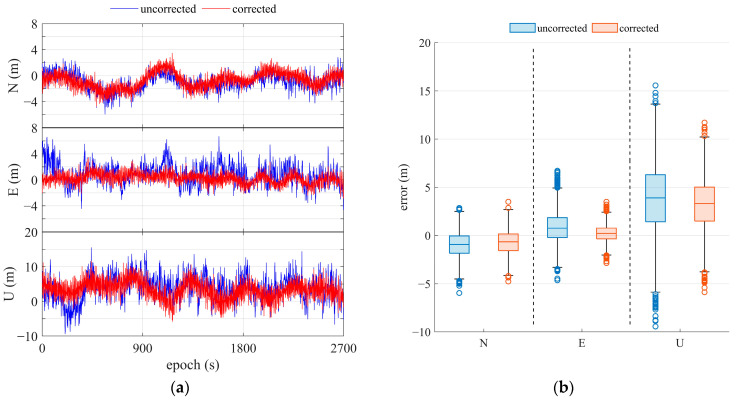
Results of single-point positioning with DRS for OEM navigation receiver. (**a**) Positioning error; (**b**) statistics of positioning error.

A static test was performed on the top floor of the geographic building at the main campus of Sun Yat-sen University, as shown in Figure 3. In comparison, two kinds of GNSS receivers are used for positioning tests and validation. One is the OEM navigation receiver, with an external compact antenna, and the other is the Huawei Mate 20 Pro receiver. Using the WT-based code multipath processing skill, the refined GNSS positioning results for the OEM navigation receiver are presented in Figure 18 and compared in Table 1. With the mitigation of code multipath in DRS, GNSS positioning accuracy is improved. The mean error in horizontal decreases by 32%, dropping from 2.12 m to 1.44 m, compared with the result without multipath correction. The mean error in vertical decreases by 15%, dropping from 3.81 m to 3.24 m, compared to the result without multipath correction. These results suggest that our proposed method can improve the accuracy of positioning affected by multipath.

**Figure 19:**
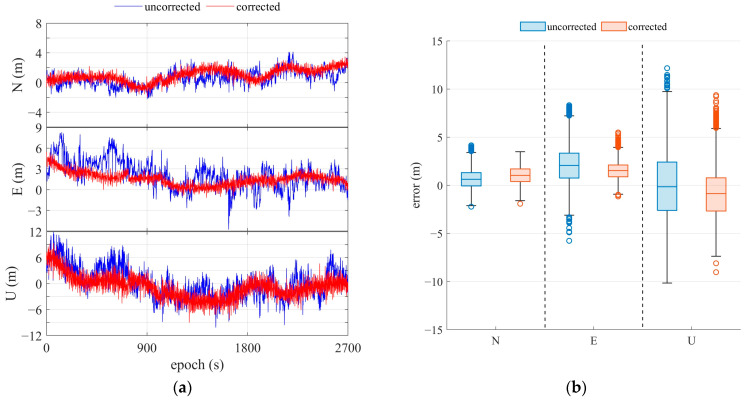
Results of single-point positioning with DRS using the Huawei Mate 20 Pro smartphone. (**a**) Positioning error; (**b**) statistics of positioning error.

The refined GNSS positioning results for the Huawei Mate 20 Pro receiver are presented in Figure 19 and compared in Table 2. From these comparisons, the improvements in positioning are obvious. After multipath mitigation and correction using WT was applied, the mean and the standard deviations of point error decreased from 4.30 m to 3.40 m and from 2.11 m to 1.49 m, respectively. The positioning accuracy improved by more than 40% and 20% in 3D and 2D horizontal positioning, respectively. Additionally, the adaptive multipath detection parameter algorithm can improve horizontal accuracy by about 15% [32]. Furthermore, it can be observed that WT-based multipath elimination can more significantly enhance smartphone positioning, as it is less effective in suppressing multipath impacts due to hardware limitations.

#### 3.3.2. Kinematic Positioning

**Figure 20:**
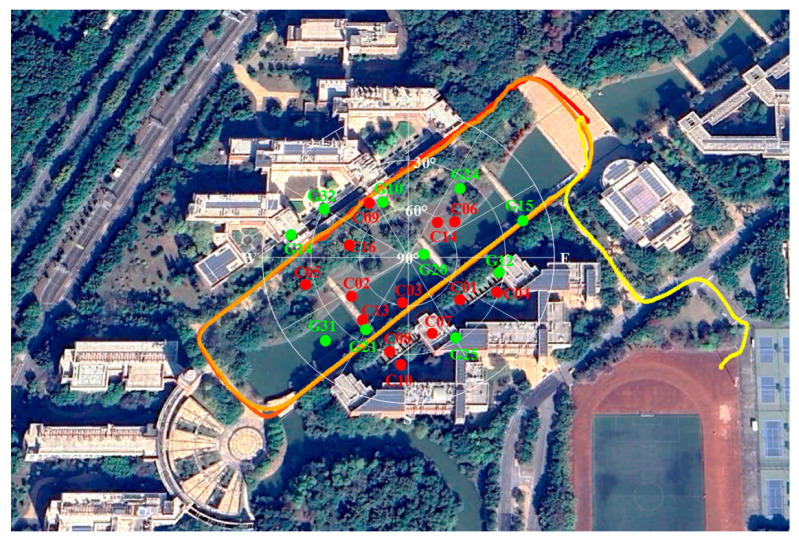
Scenario, trajectory (from red to yellow), and sky plot of satellites in kinematic positioning.

**Figure 21:**
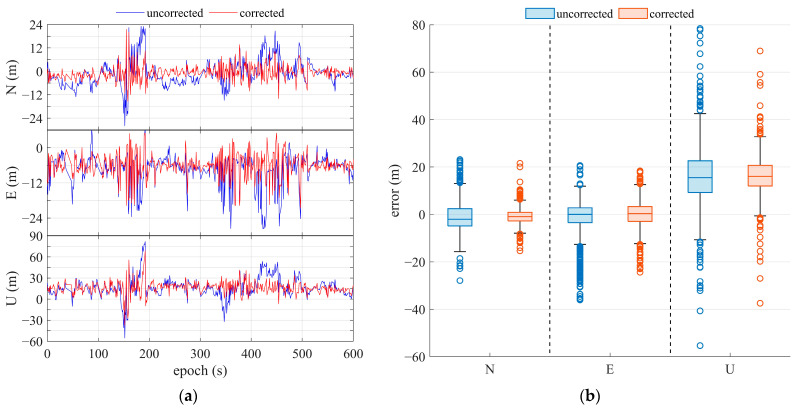
Results of kinematic positioning with DRS using the Huawei Mate 20 Pro smartphone. (**a**) Positioning error; (**b**) statistics of positioning error.

The test for kinematic positioning was conducted around buildings on the east campus of Sun Yat-sen University, where the Huawei Mate 20 Pro was used, as shown in Figure 20. The refined GNS positioning results are presented in Figure 21 and are compared in Table 3. Since signal occlusion and reflection occur in dynamic experiments, the satellite number changes, and the error in positioning increases, leading to instability compared to static positioning. The positioning accuracy was improved by more than 20% in the horizontal direction and by more than 10% in the vertical direction after the multipath correction. Meanwhile, the adaptive multipath detection parameter algorithm can improve horizontal accuracy by about 18% [32]. The max value and the standard deviation of the position error were decreased by about 20% and 40%, respectively. From these comparisons, the positioning improvements for horizontal directions are more obvious.

## 4. Conclusions and Prospects

Driven by the wide-ranging needs of LBS and positioning services, robust GNSS localization is urgently required. Code multipath as an obstinate error source in metropolitan environments is a hot target for scholars and professionals. Through comprehensive research and analysis, this study proposes a novel approach to identify, extract, and mitigate persistent multipath errors in GNSS observations using an adaptive data-driven methodology. The framework systematically employs wavelet decomposition and spectral analysis techniques to address code multipath in urban positioning scenarios. Validated by tests and experiments, the suggested technical frame fits both static and dynamic positioning.

Given the sophisticated issues involved in the research, future work is still needed to include more types of error sources in the WT-based method, such as ionosphere delay during active seasons and potential radio interferences from wireless networks. Currently, the method requires the selection of a reference satellite, resulting in a reduction in the number of satellite sources. Future tests are still needed to verify performance at lower elevation angles. Moreover, the presented technique is expected to be extended to differential positioning and precise point positioning modes in the future.

## Figures and Tables

**Table 1:** Statistics of the static positioning error with different dimensions, based on the OEM navigation receiver. For each statistic, there are two rows of data. The first row is for the result without multipath correction, and the second row is for the result with multipath correction. The percentage is the improvement of the corrected result, compared to that without correction.

Statistics	Horizontal (m)		Vertical (m)		3D Point (m)	
Max	7.13	27%	15.57	25%	15.79	24%
	5.18		11.72		12.04	
Mean	2.12	32%	3.81	15%	5.14	23%
	1.44		3.24		3.97	
RMS	1.16	28%	3.67	27%	2.72	21%
	0.83		2.68		2.16	

**Table 2:** Statistics of the static positioning error with different dimensions, based on the Huawei Mate 20 Pro. For each statistic, there are two rows of data. The first row is for the result without multipath correction, and the second row is for the result with multipath correction. The percentage is the improvement of the corrected result, compared to that without correction.

Statistics	Horizontal (m)		Vertical (m)		3D Point (m)	
Max	8.41	40%	12.16	23%	13.35	25%
	5.05		9.36		10.08	
Mean	2.80	21%	2.90	23%	4.30	21%
	2.21		2.22		3.40	
RMS	1.51	44%	3.58	22%	2.11	29%
	0.84		2.80		1.49	

**Table 3:** Statistics of the kinematic positioning error in different dimensions. For each statistic, there are two rows of data. The first row is for the result without multipath correction, and the second row is for the result with multipath correction. The percentage is the improvement of the corrected result, compared to that without correction.

Statistics	Horizontal (m)		Vertical (m)		3D Point (m)	
Max	39.88	21%	81.86	16%	87.11	17%
	31.45		68.76		72.09	
Mean	8.84	33%	18.55	10%	21.41	14%
	5.90		16.76		18.50	
RMS	8.05	36%	12.57	36%	13.64	41%
	5.12		8.06		8.05	

## Data Availability

The datasets presented in this article are not readily available because the data are part of an ongoing study. Requests to access the datasets should be directed to the corresponding author.
